# Reduced Number and Morphofunctional Change of Alveolar Macrophages in MafB Gene-Targeted Mice

**DOI:** 10.1371/journal.pone.0073963

**Published:** 2013-09-06

**Authors:** Michiko Sato-Nishiwaki, Yasuko Aida, Shuichi Abe, Yoko Shibata, Tomomi Kimura, Keiko Yamauchi, Hiroyuki Kishi, Akira Igarashi, Sumito Inoue, Masamichi Sato, Osamu Nakajima, Isao Kubota

**Affiliations:** 1 Department of Cardiology, Pulmonology and Nephrology, School of Medicine, Yamagata University, Yamagata City, Yamagata, Japan; 2 Research Laboratory for Molecular Genetics, School of Medicine, Yamagata University, Yamagata City, Yamagata, Japan; Albany Medical College, United States of America

## Abstract

Alveolar macrophages (AMs) play an important role in the pathogenesis of chronic obstructive pulmonary disease (COPD). We previously demonstrated that the transcription factor, MafB, increased in the AMs of mice exposed to cigarette smoke, and in those of human patients with COPD. The aim of this study was to evaluate the role of MafB in AMs using newly established transgenic (TG) mice that specifically express dominant negative (DN) MafB in macrophages under the control of macrophage scavenger receptor (MSR) enhancer-promoter. We performed cell differential analyses in bronchoalveolar lavage cells, morphological analyses with electron microscopy, and flow cytometry-based analyses of surface markers and a phagocytic capacity assay in macrophages. AM number in the TG mice was significantly decreased compared with wild-type (WT) mice. Morphologically, the high electron density area in the nucleus increased, the shape of pseudopods on the AMs was altered, and actin filament was less localized in the pseudopods of AMs of TG mice, compared with WT mice. The expression of surface markers, F4/80 and CD11b, on peritoneal macrophages in TG mice was reduced compared with WT mice, while those on AMs remained unchanged. Phagocytic capacity was decreased in AMs from TG mice, compared with WT mice. In conclusion, MafB regulates the phenotype of macrophages with respect to the number of alveolar macrophages, the nuclear compartment, cellular shape, surface marker expression, and phagocytic function. MSR-DN MafB TG mice may present a useful model to clarify the precise role of MafB in macrophages.

## Introduction

Chronic obstructive pulmonary disease (COPD) is a major cause of chronic morbidity and mortality throughout the world [1]. Importantly, COPD is increasingly a cause of chronic disability and is predicted to become the fifth most common cause of chronic disability, worldwide, by 2020. The pathophysiology of COPD is thought to involve chronic inflammation, with increased numbers of specific inflammatory cells and parenchymal destruction [2]. The numbers of alveolar macrophages (AMs) significantly increase in the lungs of COPD patients [3]. AMs play important roles in the pathogenesis and pathophysiology of COPD, and may account for most of the known features of the disease [4,5].

We previously demonstrated that the transcription factor MafB was upregulated in AMs in the lungs of mice with cigarette smoke-induced pulmonary emphysema. In addition, overexpression of MafB enhanced cell viability and attenuated apoptosis in cells treated with cigarette smoke extract [6]. Moreover, we reported that the intensity of immunostaining of MafB in AMs correlated with the degree of airflow limitation in human smokers [7]. MafB is selectively expressed in monocytes and macrophages but not in other haematopoietic cells [8], and it induces monocyte differentiation [9].

The Maf protein family possesses a basic leucine zipper structure at the carboxyl terminus, which mediates dimer formation and DNA binding to the Maf recognition element (MARE) [10]. Toward the N-terminal of the Maf protein, there is an acidic domain that can transactivate the transcription of MARE regulated genes. A truncated form of MafB lacking the N-terminal acidic domain reportedly acts as a dominant negative (DN) [9]. DN MafB forms heterodimers with endogenous MafB and inhibits binding of endogenous MafB homodimers with MARE.

MafB knockout mice die immediately after birth due to developmental anomalies of neurons in the respiratory center [11,12]. In addition, MafB homozygous mutants display renal dysgenesis with abnormal podocyte differentiation, hypoplasia of the inner ears, and developmental and functional disorder of the macrophages [13]. Thus, the role of MafB in the pathogenesis of various chronic diseases, including COPD, have not been fully investigated *in vivo* due to the lack of an adequate animal model, and an advanced gene targeted animal is required to reveal the exact role of MafB.

Recent developments in gene-targeting technology have enabled us to regulate tissue-specific gene expression, including the DN protein form. Jin et al. generated a mouse that expressed the DN form of transcription factor ets-2 under the control of macrophage-colony stimulating factor (M-CSF) receptor proximal promoter [14]. In this mouse, the phenotype of the peritoneal macrophages was altered compared with those in the control mouse. Horvai et al. demonstrated that the macrophage scavenger receptor (MSR) enhancer-promoter was useful for macrophage specific gene expression [15].

We sought to clarify the role of MafB in macrophages *in vivo* using newly engineered transgenic (TG) mice (MSR DN MafB TG mice), and aimed to evaluate the phenotype of these mice. Some of the results presented in this manuscript have been previously reported in abstract form [16].

## Methods

### Making of the human macrophage scavenger receptor (MSR) enhancer-promoter construct

**Figure 1:**
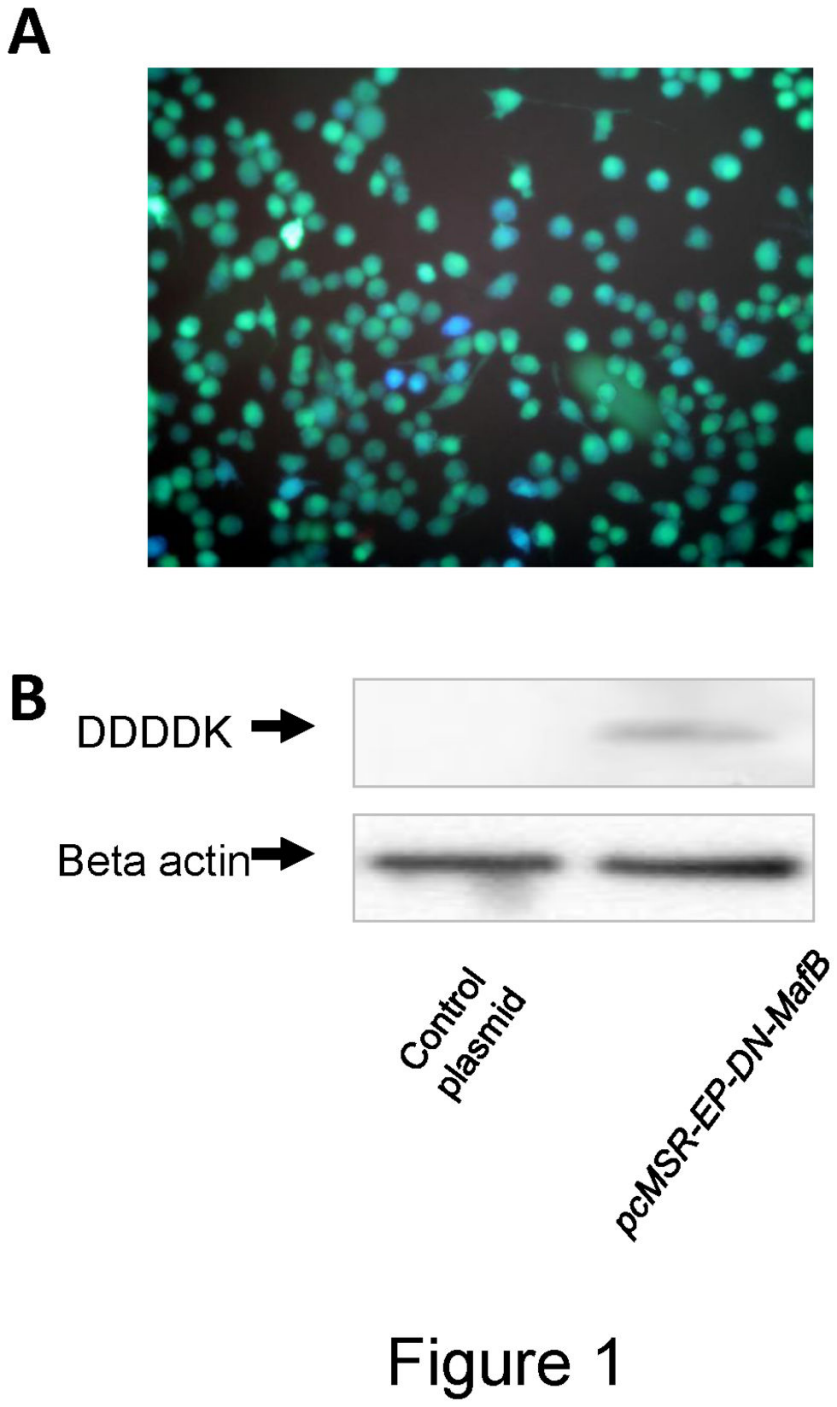
Verification of the plasmid vector containing human macrophage scavenger receptor (MSR) enhancer-promoter and the vector that enables expression of dominant negative (DN) MafB under the control of the MSR enhancer-promoter. (A) The reporter plasmid containing the MSR enhancer-promoter (*pMSR-EP-Bla*) was transfected into the RAW264.7 macrophage cell line, and reporter activity was assessed as described in the *Materials and Methods* in the online supplement. Positive activity of MSR enhancer-promoter was confirmed by the presence of ‘blue cells’ (as indicated in the photograph). (B) *pcMSR-EP-DN-MafB* and empty control plasmid were transfected into RAW264.7 cells. DN MafB protein in cell homogenates was evaluated by immunoblotting using a DDDDK-tag antibody. As an internal control, the amount of beta actin was also assessed. A 15 KDa band, estimated size of DN MafB, was only detected in RAW264.7 cells transfected with *pcMSR-EP-DN-MafB*.

MSR enhancer and promoter regions were amplified from human genomic DNA templates of healthy volunteers by polymerase chain reaction (PCR) using Platinum Taq DNA polymerase (Invitrogen, Corp., CA, USA) and specific primers (enhancer sense: tgc agg aga cag ctg atc ttg caa gga aat tag, enhancer antisense: cag tcc ggg gac aag ggc cca cta aaa gac tga, promoter sense: gct gta aat tat gtg ctt gtt tca aca ac, promoter antisense: cct aaa gaa agc agc act gat tta tcc act) [15]. After confirming the PCR product size by electrophoresis (enhancer, 422 bp and promoter, 291 bp), both PCR products were mixed and amplified by PCR using primers (enhancer sense, promoter antisense, and linking primer: aca agc aca taa ttt aca gcc agt ccg ggg aca agg gcc c). This resulted in a product of 713 bp, equivalent to the summarized size of the MSR enhancer with promoter. This DNA fragment was subcloned into pGeneBLAzer-TOPO (Invitrogen) and the resultant plasmid vector was called *pMSR-EP-Bla*. The sequence of *pMSR-EP-Bla* was confirmed using the ABI PRISM 3130 Genetic Analyzer. This plasmid vector was transfected into RAW264.7 cells using GenePORTER2 (Gene Therapy Systems Inc., San Diego, CA), as per manufacturer’s instructions. We confirmed the reporter activity induced by the MSR enhancer-promoter of *pMSR-EP-Bla* in a macrophage cell line by using the GeneBLAzer *in vivo* detection kit (Invitrogen) (Figure 1A) [17]. Transfected cells were incubated with CCF2-AM, a substrate of β-lactamase, for 1 hour, and subject to fluorescent microscopy (BX63; Olympus, Japan). Uncleaved CCF2-AM substrate has green fluorescence but when cleaved by β-lactamase it changes to blue fluorescence [18].

### Making the murine dominant negative (DN) MafB plasmid

The truncated form of MafB protein, amino acids 191 to 323, is suggested to act as a DN due to the lack of a transactivation domain [9]. A DNA fragment equivalent to the DN MafB region was amplified from mouse genomic DNA using Accuprime Pfx DNA polymerase (Invitrogen) using specific primers (sense: cac cat ggg acc gca cgc aac agc cgc ggc, antisense: cta tta ctt gtc atc gtc atc ctt ata gtc gcc aga acc cag aaa gaa ctc agg aga gga ggg gct g). The DDDDK tag sequence was artificially inserted in the antisense primer. After confirming the size of the amplified fragment (450 bp), the PCR product was subcloned into pcDNA6.2/V5/GW/D-TOPO (Invitrogen) as per the manufacturer’s instructions, to give rise to *pcDN-MafB*. The sequence of *pcDN-MafB* was confirmed with the ABI PRISM 3130 Genetic Analyzer.

### Combining the MSR enhancer-promoter with DN MafB

The cytomegalovirus promoter in *pcDN-MafB* was removed and linearized using restriction enzymes, *Kpn1* and *Pst1*, (*pcDN-MafB-CMV-minus*). The MSR enhancer-promoter DNA fragment was amplified from *pMSR-EP-Bla* using specific primers containing the *Kpn1* and *Pst1* restriction sites (sense containing *Kpn1* restriction site; cag tgg ggt acc tgc agg aga cag ctg atc ttg caa gga aat tag, antisense containing *Pst1* restriction site: ggt ggc ctg cag cct aaa gaa agc agc act gat tta tcc act). The resultant DNA fragment was treated with *Kpn1* and *Pst1*, and ligated with *pcDN-MafB-CMV-minus* using Ligation Mighty (Takara Bio, Shiga, Japan) giving rise to *pcMSR-EP-DN-MafB*. To confirm DN MafB expression by this plasmid, *pcMSR-EP-DN-MafB* was transfected into RAW264.7 cells using GenePORTER2 (Gene Therapy Systems), as per manufacturer’s instructions. Whole cell lysate (50 µg) was used for immunoblotting assays with mouse DDDDK antibody (1:500, Sigma, MO, USA), and anti-mouse IgG (1:5000, Promega Corp., WI, USA). A specific 15 kDa-band was detected only in *pcMSR-EP-DN-MafB*-transfected RAW264.7 cells, compatible with the size of DN MafB (Figure 1B).

### Generating MSR-DN MafB transgenic (TG) mice

To express DN MafB specifically in macrophages *in vivo*, we used *pcMSR-EP-DN-MafB* to generate MSR-DN MafB TG mice. The *pcMSR-EP-DN-MafB* plasmid was digested with BglII to generate 1.9 kb and 3.8 kb linear fragments, and microinjected into the pronuclei of fertilized C57BL/6 mouse eggs, which were implanted into a foster mouse to generate a pseudopregnant. To detect the exogenous DN MafB gene, genomic DNA was extracted from the tail tissue of 4-week-old mice, and PCR with the sense (ggg ccc tct aga tca acc ac) and antisense (acg cct aca agg tca agt gc) primers, was performed. Finally, MSR-DN MafB mice were generated, and age-matched non-TG littermates and wild-type (WT) mice were used for the study.

Mice were housed in a facility with a 12/12-h light/dark cycle and were given free access to water and standard rodent chow. The rooms were kept free from any pathogens. All mice were handled according to a guide for the care and use of laboratory animals at Yamagata University, and the study protocol was approved by the Animal Subjects Committee of Yamagata University. The investigation conformed to the *Guide for the Care and Use of Laboratory Animals* published by the US National Institutes of Health. The general appearance of the TG mice was not obviously different to the wild-type (WT) or non-TG littermates. No neonatal death was observed in the TG mice, and their survival rate was similar to the non-TG littermates (data not shown).

### Bronchoalveolar lavage (BAL) and harvesting of AMs

Mice were anesthetized and a 20-gauge catheter was inserted into the trachea. BAL was performed by instilling 1 mL of phosphate buffered saline (PBS) six times with a syringe. The total numbers of BAL cells were counted under a light microscope using a hemacytometer. Some BAL cells were fixed on slides by subjecting the BAL to a cytospin of 1000 rpm for 5 minutes, and these were stained with a Diff-Quick solution (International Reagents Corp., Kobe, Japan) for differential cell counts. Remaining BAL cells were incubated with Dulbecco’s Modified Eagle Medium (DMEM, Invitrogen) supplemented with 20% fetal bovine serum, 1% antimyconic (Invitrogen), and L-Glutamine (TaKaRa) in a culture dish at 37 °C for 1 hour. AMs were obtained as attached cells on the dish.

### Harvesting peritoneal macrophages (PMs)

Mice were anesthetized and injected intraperitoneally with 2 mL of a 3% Brewer’s thioglycollate (BD Bioscience, MD, USA) solution. After five days, mice were sacrificed and peritoneal cells were collected by lavage with 5 mL PBS. The lavage fluid was centrifuged, and the cell pellet was resuspended in RPMI1640 medium (Invitrogen). Cells were subjected to PCR or flow cytometric analysis.

### Harvesting bone marrow-derived macrophages (BMDMs)

Bone marrow cells were harvested from femoral and iliac bones. Cells were cultured in a flask with DMEM as described above, and incubated with 10 ng/mL murine macrophage-colony stimulating factor (M-CSF) (R&D systems, Minneapolis, USA). Culture medium was replaced every second day and BMDMs were obtained after seven days incubation. For the reverse transcription (RT)-PCR analysis of DN-MafB and MSR in bone mallow hematopoietic progenitor cells, the concentration of bone mallow cells were adjusted to 1 × 10^8^ cells/mL in PBS. Then bone mallow hematopoietic progenitor cells were isolated using EasySep^TM^ Mouse Hematopoietic Progenitor Enrichment Kit (STEMCELL^TM^ Technologies, Vancouver, Canada) according to the manufacturer’s protocol. Then, cells were differentiated by murine M-CSF treatment in DMEM for 7days.

### Isolation of RNA and RT-PCR

Total RNA was isolated from macrophages or liver tissues using an RNeasy Mini Kit (QIAGEN, Valencia, CA, USA), according to the manufacturer’s protocol. RT was performed using SuperScript III (Invitrogen) and gene expression was assessed by RT-PCR. The sequences of the specific primers were: caspase-3 forward, CTC GCT CTG GTA CGG ATG TG; reverse, AGT TCA ACA GGC CCA TTT GTC; DN MafB forward, ACG CCT ACA AGG TCA AGT GC; reverse, GGG CCC TCT AGA TCA ACC AC; MafB forward, CGT CCA GCA GAA ACA TCA CC; reverse, TCG CAC TTG ACC TTG TAG GC; GAPDH forward, CTT CAC CAC CAT GGA GAA GGC; reverse, GGC ATG GAC TGT GGT CAT GAG; MSR forward, AAG TTG AAG TCC TTC AAG GCT GCC; reverse, GCA TCC AGT GAA TTC CCA TGT TCC. PCR products were electrophoresed in 1.5% agarose gels containing ethidium bromide and visualized digitally on a UV illuminator (AB1500 Printgraph and AE 6905H Image Saver HR; ATTO Bioscience, Tokyo, Japan). The band intensities were semi-quantified using computer software (Lane Analyzer 3.0; ATTO Bioscience).

### Immunohistochemistry of DDDDK tag and Mac3

Mice were sacrificed under deep anesthesia by intraperitoneal injection of 10% pentobarbital. The lungs were excised and fixed intratracheally with 4% paraformaldehyde at a constant pressure of 25 cm H_2_O to prepare the paraffin-embedded lung blocks. Other tissues were also fixed with 4% paraformaldehyde. Paraffin embedded tissue sections were cut at 3-µm thickness and placed on glass slides. Sections were incubated with goat DDDDK tag antibody (1:500, Abcam Plc, Cambridge, UK), followed by anti-goat IgG-HRP (1:200, Santa Cruz Biotechnology Inc., CA, USA). Color development was performed using DAKO DAB substrate-chromogen system (DAKO, Japan). Nuclei were counterstained with hematoxylin. All tissue sections were simultaneously stained to avoid technical variances due to differences in staining conditions. No positive IHC staining was preliminarily observed in the negative control. For Mac-3 staining, cytospun BAL cells were used. BAL cells were fixed with an acetone/methanol (1:1) solution, and then incubated with Mac-3 antibody (1:100, Becton, Dickinson and company), and anti-rat IgG-HRP (1:200, Stressgen, CA, USA). Nuclei were counterstained with hematoxylin.

### Immunofluorescence staining of DDDDK tag and Mac-3

Paraffin embedded lung tissue sections were cut at 3-µm thickness and placed on glass slides. Sections were incubated with a goat DDDDK tag antibody (1:1000, Abcam Plc) followed by PE labeled-anti-goat IgG (1:200, Santa Cruz), Mac-3 antibody (1:1000, BD Pharmingen, NJ, USA), and anti-rat IgG-FITC (1:100, VECTOR Laboratories, Inc., CA, USA). No positive immunofluorescence staining was preliminarily observed in the negative control. Samples were observed under a fluorescence microscope (DMI3000B; Leica, Solms, Germany).

### Analysis of apoptosis

Caspase-3 RT-PCR and TdT-mediated dUTP-biotin nick end labeling (TUNEL) were employed to evaluate apoptosis in macrophages in TG mice. Caspase-3 gene expression in AMs was evaluated as described above. For TUNEL staining, paraffin embedded tissue sections were cut at 3-µm thickness and placed on glass slides. Sections were stained using the *In Situ* Cell Death Detection Kit, Fluorescein (Roche, Basel, Switzerland), according to the manufacturer’s protocol. No positive immunofluorescence staining was preliminarily observed in the negative control. Samples were observed by fluorescence microscopy (DMI3000B; Leica).

### Flow Cytometry

Cell surface markers (F4/80 and CD11b) of AMs and thioglycollate-elicited PMs were analyzed by flow cytometry. AMs or PMs were resuspended in fluorescence-activated cell sorter (FACS) buffer (PBS, 0.2% BSA, 0.01% sodium azide). Erythrocytes were hemolyzed before assessing. AMs and PMs were stained with FITC-labeled F4/80 antibody (AbD serotec, Kidlington, UK), FITC-labeled CD11b antibody (BD Pharmingen), or isotype control (BD Pharmingen) at 4°C for 30 minutes. Stained cells were washed in FACS buffer and subjected to flow cytometry using the FACS Calibur or FACS Canto II (BD Biosciences). Data were analyzed using CellQuest or DIVA software (BD Bioimaging systems, MD, USA). The mean fluorescence index (MFI) was calculated with the following formula: The mean fluorescence index = the percentage of gated cells × mean fluorescence intensity.

### Transmission electron microscopy (TEM) and scanning electron microscopy (SEM) analysis of AMs

AMs were obtained from BAL fluid and fixed in 2.5% glutaraldehyde at room temperature for 7 days. After fixation for 1.5 h with 2% osmium tetroxide in phosphate buffer, cells were dehydrated in ethanol and embedded in epoxy resin. Epoxy resin embedded sections were cut at 0.5-µm thickness and placed on glass slides. Sections were stained with 1% toluidine blue to find appropriate areas for sectioning. The ultrathin sections were cut at 50-nm thickness and stained with 2% uranyl and lead solution, and observed with the Hitachi H-7100 electron microscope (Hitachi, Tokyo, Japan). Digital images were taken with the Megaview β digital camera (Seika Corp., Tokyo, Japan), and 80 nuclei, which had adequate cross-sections, were randomly selected from each subject. Edges on the images of the whole nucleus and the high electron density region within the nucleus were traced using computer software (ImageJ) to obtain the area. Fractions of high-density area in the nuclei were calculated, and compared between MSR-DN MafB TG mice and WT mice. The cell surface morphology of AMs was evaluated with scanning electron microscopy (Hitachi S-5000S electron).

### Confocal microscopy of actin filament

AMs were harvested into cover-glass chambers (Iwaki Garasu, Japan), and cultured in DMEM with 20% bovine serum for 37°C and 5% CO2 for 24 h. After removing unattached cells by gentle medium exchange, cells were fixed with 4% paraformaldehyde. Following staining with Alexa Fluor 488 Phalloidin (1:40, Invitrogen) cells were observed using a LSM-510 Meta (Carl Zeiss, Germany).

### Measurement of Rho GTPase activity

Rho GTPase activity was measured using a commercially available G-LISA RhoA Activation Assay Kit (Cytoskeleton, Inc, Denver, CO), according to the manufacturer’s instructions. Briefly, 3 x 10^s^ BMDMs of WT and MSR-DN MafB TG mice were harvested onto a culture dish, and cultured in DMEM containing 20% bovine serum and 10 ng/mL M-CSF at 37°C and 5% CO2 for seven days. After homogenization of the cells, the lysates were put into a Rho-GTP-binding plate. The homogenates were incubated with secondary antibody HRP (1:250), and Rho-GTPase activity in the homogenate was measured using a Varioskan flash multispectro-microplate reader (Thermoscientific).

### β-lactamase Reporter assay

To evaluate whether transduced DN MafB had less transcriptional binding activity to MARE, β-lactamase reporter activity was assayed. The plasmid *pMARE-Bla* [6], which contains trimerized MAREs and β-lactamase reporter genes, was transfected into 1x10^6^ BMDMs from WT and DN MafB TG mice using the Nucleofector electroporation system (Amaxa, Lonza Group Ltd, Switzerland), as per the manufacturer’s protocol. Transfected BMDMs were cultured in RepCell dishes (Cell Seed, Tokyo, Japan) with DMEM containing 20 µM 4-hydroxy-2-nonenal (4-HNE) for 24 hours. β-lactamase activity was measured using the GeneBLAzer *in vivo* detection kit (Invitrogen Corp., CA, USA) [17]. BMDMs were incubated with CCF2-AM, a β-lactamase substrate, for 1 hour, and subject to flow cytometry (FACS Canto II; BD Biosciences, CA, USA). Intact CCF2-AM substrate has green fluorescence and when it is cleaved by β-lactamase, it changes into blue fluorescence [18]. . Data are indicated as the number of β-lactamase positive cells per 10,000 cells.

### Flow-based Fc receptor mediated phagocytosis assay

Phycoerythrin (PE)-labeled fluorescent beads (Spherotech, Libertyville, IL, USA) were pre-coated with bovine serum albumin (BSA). IgG-opsonized fluorescent beads were prepared by incubating BSA-coated beads with anti-albumin antibody (Sigma, MO, USA) followed by washing and resuspension. AMs were collected from BAL fluid, and were exposed to IgG-opsonized beads at a concentration of 5 × 10^6^ beads per 1 × 10^6^ cells in 1 mL culture medium for 1 hour. Cells were washed in PBS to remove non-internalized particles, detached by trypsinization, and evaluated by flow cytometry using FACS Canto II (BD Biosciences). The phagocytic index was calculated with the following formula: phagocytic index = percent of cells containing beads × mean fluorescence of cells containing beads [19].

### Statistical analyses

Results are expressed as the mean ± standard deviation. Significance was inferred for *P* values < 0.05. Statistical analyses were performed using StatView - J 5.0 computer software (SAS Institute, Inc., Cary, NC, USA).

## Results

### DN MafB mRNA and protein expression in macrophages of MSR-DN MafB TG mice

**Figure 2:**
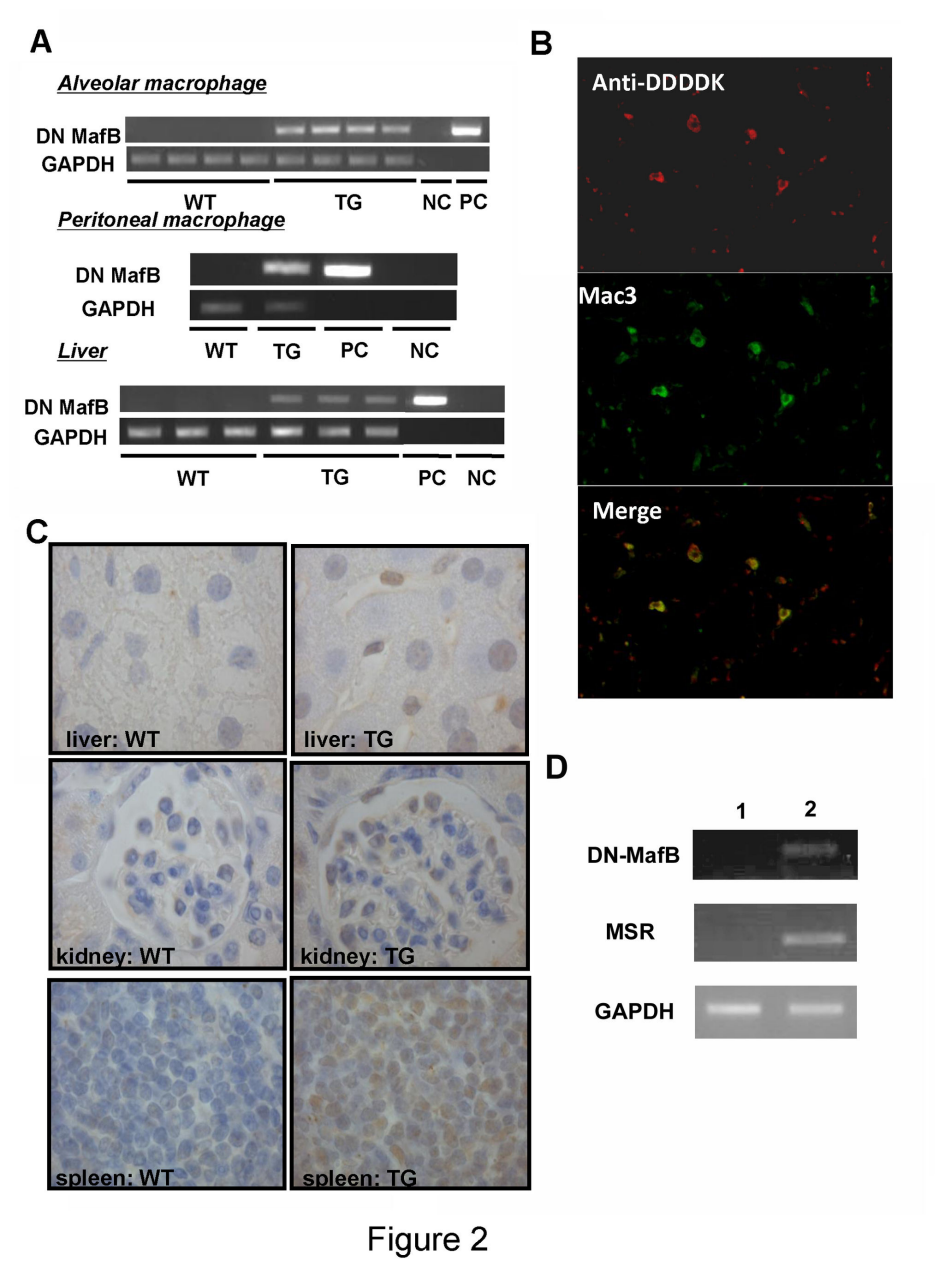
Verification of mice expressing dominant negative (DN) MafB only in macrophages under the control of the human macrophage scavenger receptor (MSR) enhancer-promoter. (A) DN MafB mRNA expression was confirmed by RT-PCR using specific primers between the MafB sequence and the DDDDK tag sequence. DN MafB mRNA was positive in alveolar macrophages, peritoneal macrophages and the liver of transgenic (TG) mice, but negative in all of these in wild-type (WT) mice. (B) The lungs of TG mice were double stained with a DDDDK tag antibody (top panel: phycoerythrin, red) and a Mac-3 antibody (center panel: FITC, green). As shown in the bottom panel, DDDDK-tag positive cells and Mac-3 positive cells were merged, indicating that alveolar macrophages were positive for DN MafB. In the lungs of WT mice, DDDDK tag positive cells were not observed (data not shown). Original magnification: ×1000. (C) DDDDK-tag was immunostained in the liver, kidney, and spleen of TG and WT mice. Positive cells for DDDDK were only confirmed in the liver, kidney and spleen of MSR-DN MafB TG mice. Original magnification: ×1000. Counterstain: haematoxylin. (D) RT-PCR. In hematopoietic progenitor cells, neither DN-MafB (top) nor MSR (middle) mRNA expressions were detected (lane 1). After M-CSF treatment for 7 days, both DN-MafB and MSR were expressed in the macrophages derived from hematopoietic progenitor cells (lane2). GAPDH (bottom): internal control.

DN MafB mRNA expression in AMs, PMs and the liver of TG mice was confirmed by RT-PCR. DN MafB expression was detected in TG mice but not in WT mice (Figure 2A). DN MafB protein production in AMs was assessed by immunofluorescent staining. The DDDDK tag was positive in cells expressing Mac-3, the specific macrophage marker, in the lungs of TG mice (Figure 2B), but not WT mice (data not shown). Immunohistochemical staining of the DDDDK tag was performed in liver, kidney, and spleen tissues of WT and TG mice. Kupffer cells in the liver, podocytes and Bowman capsule cells in the kidney, and splenic red pulp cells in the spleen were positive for the DDDDK tag (Figure 2C). To confirm whether DN MafB was expressed under the control of MSR enhancer-promoter, we evaluated the simultaneous increase of DN MafB and MSR gene expressions during the differentiation from bone marrow hematopoietic progenitor cells to macrophages cultured in M-CSF ex vivo in TG mice. The expression of MSR and DN-MafB was not detected in bone mallow hematopoietic progenitor cells. After M-CSF treatment, both MSR and DN-MafB were expressed in the differentiated macrophages (Figure 2D).

### Repression of MafB transactivation potential by DN MafB

**Figure 3:**
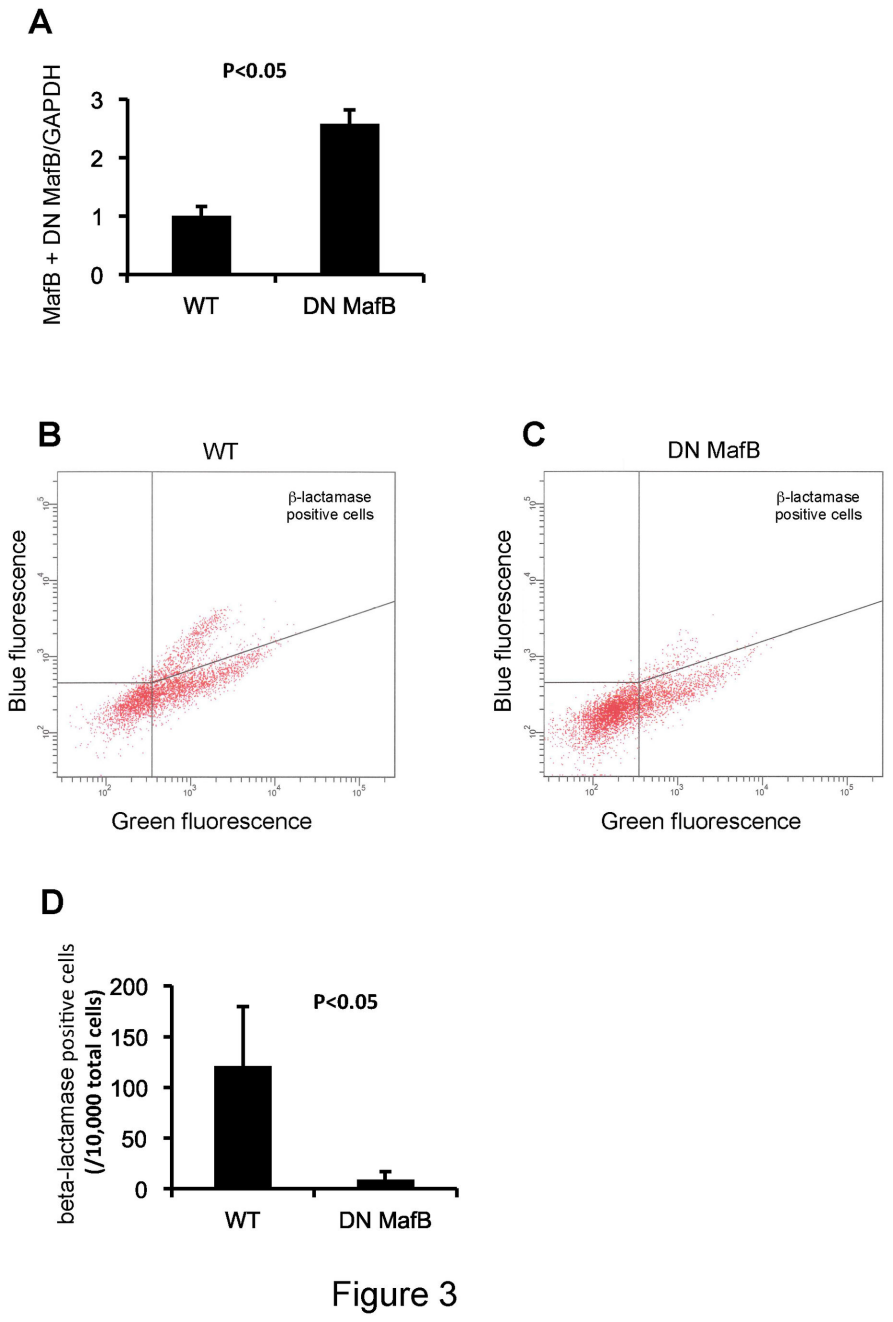
Repression of MafB activity in macrophages of macrophage scavenger receptor (MSR) enhancer-promoter dominant negative (DN) MafB transgenic (TG) mice. (A) The total amount of MafB plus DN MafB gene expression was assessed by RT-PCR in wild-type (WT) mice and TG mice. A DN MafB primer set that could amplify both DN MafB and endogenous MafB mRNA was used. PCR products were significantly increased in TG mice, compared with WT mice (n = 6 for both). (B and C) Bone marrow derived macrophages (BMDMs) from TG and WT mice were stimulated with 4-HNE to enhance endogenous MafB expression. Subsequently, BMDMs were transfected with control vector or 3× Maf recognition element (MARE) reporter plasmid vector. MafB expression was confirmed with a β-lactamase reporter assay using flow cytometry (n = 6 in each group). Dot plot data of both WT and TG BMDMs were shown in Figure 3B and 3C, respectively. The β-lactamase-positive cells had blue fluorescence. (D) The numbers of β-lactamase-positive cells were significantly lower in BMDMs of TG than those of WT.

To confirm whether the amount of DN MafB was enough to suppress endogenous MafB, RT-PCR was performed using DN MafB primers to amplify both DN MafB and endogenous MafB mRNA. As shown in Figure 3A, about 2.5 times MafB plus DN MafB mRNA was expressed in the AMs of TG mice, compared with WT mice. We previously demonstrated that MafB expression was increased by 4-HNE in a murine macrophage cell line [6]. To evaluate the alteration of endogenous MafB activity, a β-lactamase reporter assay was performed in the presence of 4-HNE. As shown in Figure 3B–D, β-lactamase-positive cells were observed in BMDMs from WT mice and were significantly decreased in BMDMs from TG mice.

### BAL cells in DN MafB TG mice

**Figure 4:**
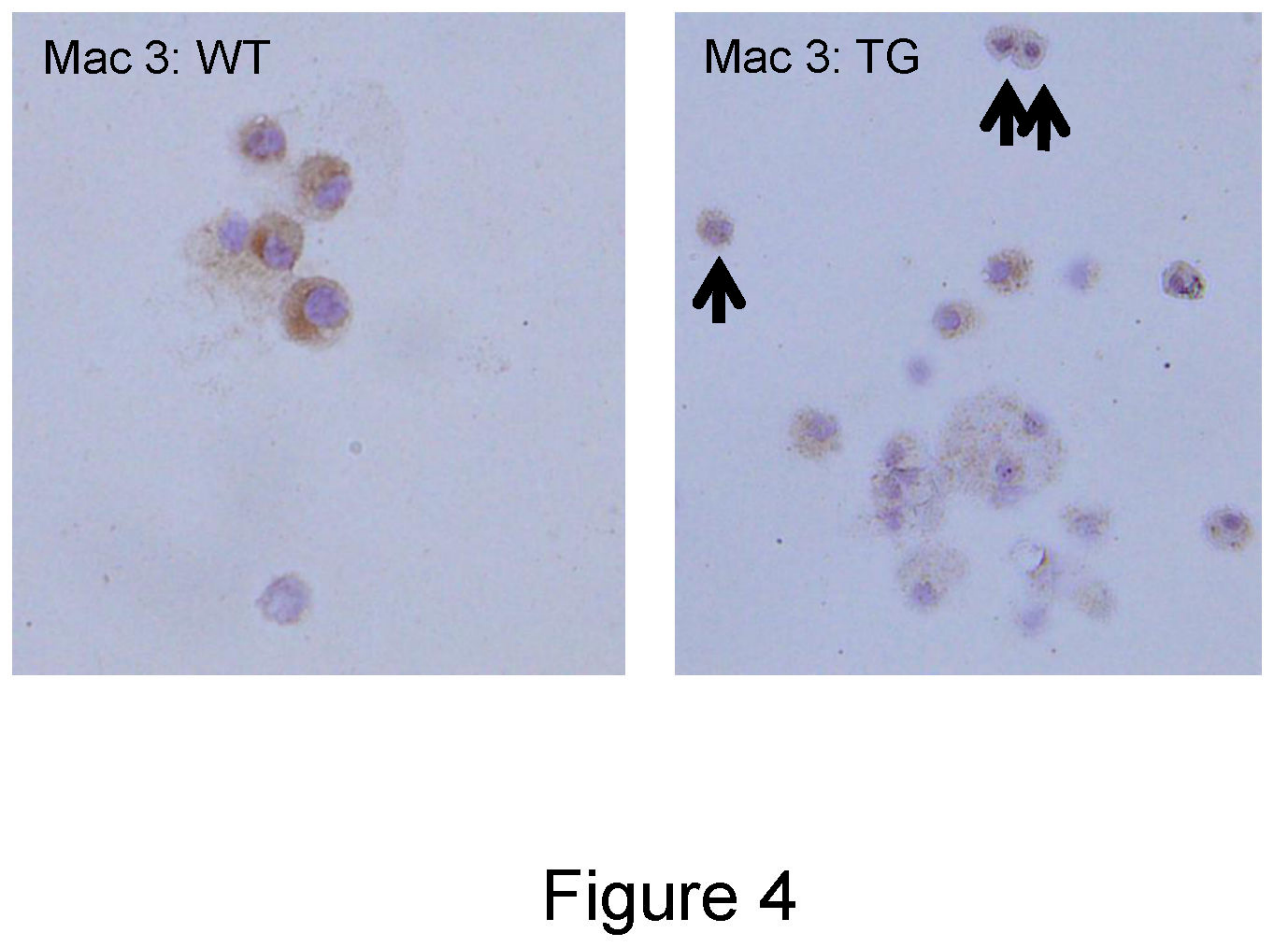
An increase in alveolar monocyte-like cells in macrophage scavenger receptor (MSR)-dominant negative (DN) MafB transgenic mice. In BAL cells from MSR-DN MafB transgenic mice, mononuclear cells were frequently observed compared with wild-type (WT) mice (arrow). Their cytoplasmic granularities were less than alveolar macrophages, but they had pseudopods on the cell surface, a typical finding for phagocytes. They were also positive for Mac-3, suggesting that they were monocytic lineage cells, namely alveolar monocyte. Original magnification: ×1000.

The concentration of BAL cells in the TG mice was significantly decreased compared with WT mice. The number of macrophages and neutrophils were decreased in TG mice (Table 1). In addition, the percentage of macrophages in BAL was significantly decreased in TG mice compared with WT mice (Table 1). In BAL, ‘mononuclear cells’ that had pseudopods (typical of phagocytes) on the cell surface and were positive for Mac-3 (Figure 4), were frequently observed in TG mice, compared with WT mice (Table 1). These cells could not be identified as differentiated macrophages due to their smaller cellular size and lesser cytoplasmic granularity. Based on morphological findings and their positivity for Mac-3, these cells were suggested to be a premature form of monocytic lineage cells, namely alveolar monocytes.

**Table 1:** Comparison of BAL fluid in wild-type and dominant negative (DN) MafB transgenic mice.

	Wild-type n=17	DN MafB n=21
Weight (g)	23.3 ± 3.5	22.1 ± 3.5
BALF volume (mL)	5.3 ± 0.3	5.3 ± 0.3
Cell concentration (×10^4^ /mL)	2.0 ± 0.6	1.2 ± 0.5**
*Cell fractionation* (×10^2^/mL)		
Macrophage	186.6 ± 59.3	107.0 ± 42.9**
Neutrophil	1.4 ± 1.4	0.4 ± 0.7*
Lymphocyte	1.9 ± 2.0	1.1 ± 0.3
Eosinophil	0.3 ± 1.0	0.0 ± 0.0
Mononuclear cells	6.6 ± 3.6	7.1 ± 4.7
*Cell fractionation (%)*		
Macrophage	95.8 ± 2.3	91.4 ± 4.0*
Neutrophil	0.1 ± 0.3	0.1 ± 0.3
Lymphocyte	2.2 ± 1.4	1.9 ± 1.2
Eosinophil	0.0 ± 0.0	0.0 ± 0.1
Mononuclear cells	1.8 ± 1.2	6.6 ± 3.2**

^*^ P < 0.01, ^**^** P < 0.0001 versus wild-type mice

### Analysis of apoptosis in the AMs and spleen of MSR-DN MafB TG mice

**Figure 5:**
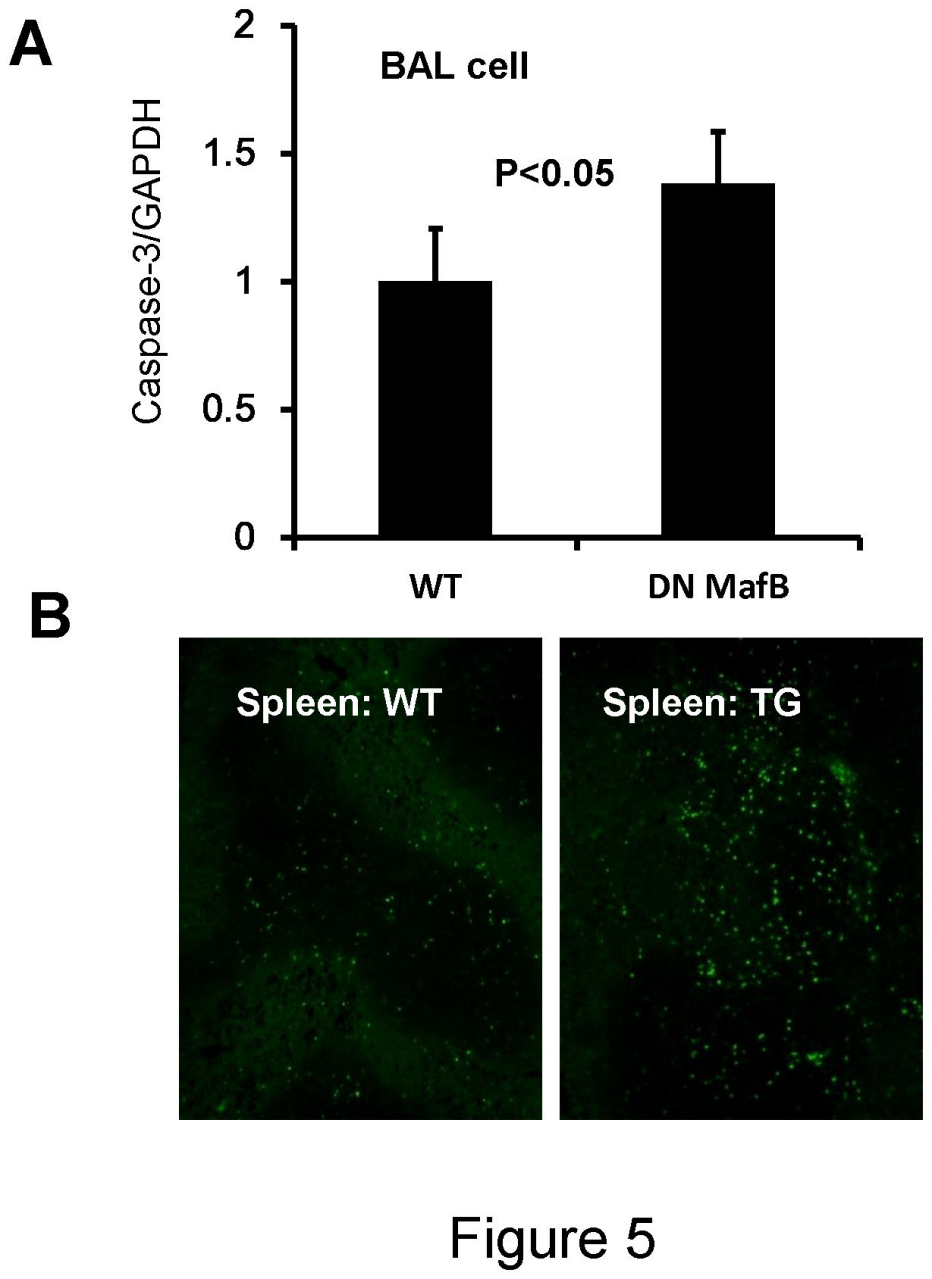
Increased apoptosis in macrophages of macrophage scavenger receptor (MSR) enhancer-promoter dominant negative (DN) MafB transgenic (TG) mice. (A) RT-PCR: Expression of caspase-3 was significantly increased in BAL cells of TG mice compared with WT mice (n = 6 for both). (B) TUNEL staining: TUNEL positive cells were increased in the spleen of TG mice, compared with WT mice. Original magnification: ×100.

To assess whether apoptosis was responsible for the reduced number of AMs, we examined the expression of caspase-3 and TUNEL positive cells in the spleen. Caspase-3 expression was significantly increased in BAL cells of TG mice compared with WT mice (Figure 5A). TUNEL positive cells were increased in the spleen of TG mice (Figure 5B).

### Morphological changes in the AMs from MSR-DN MafB TG mice

**Figure 6:**
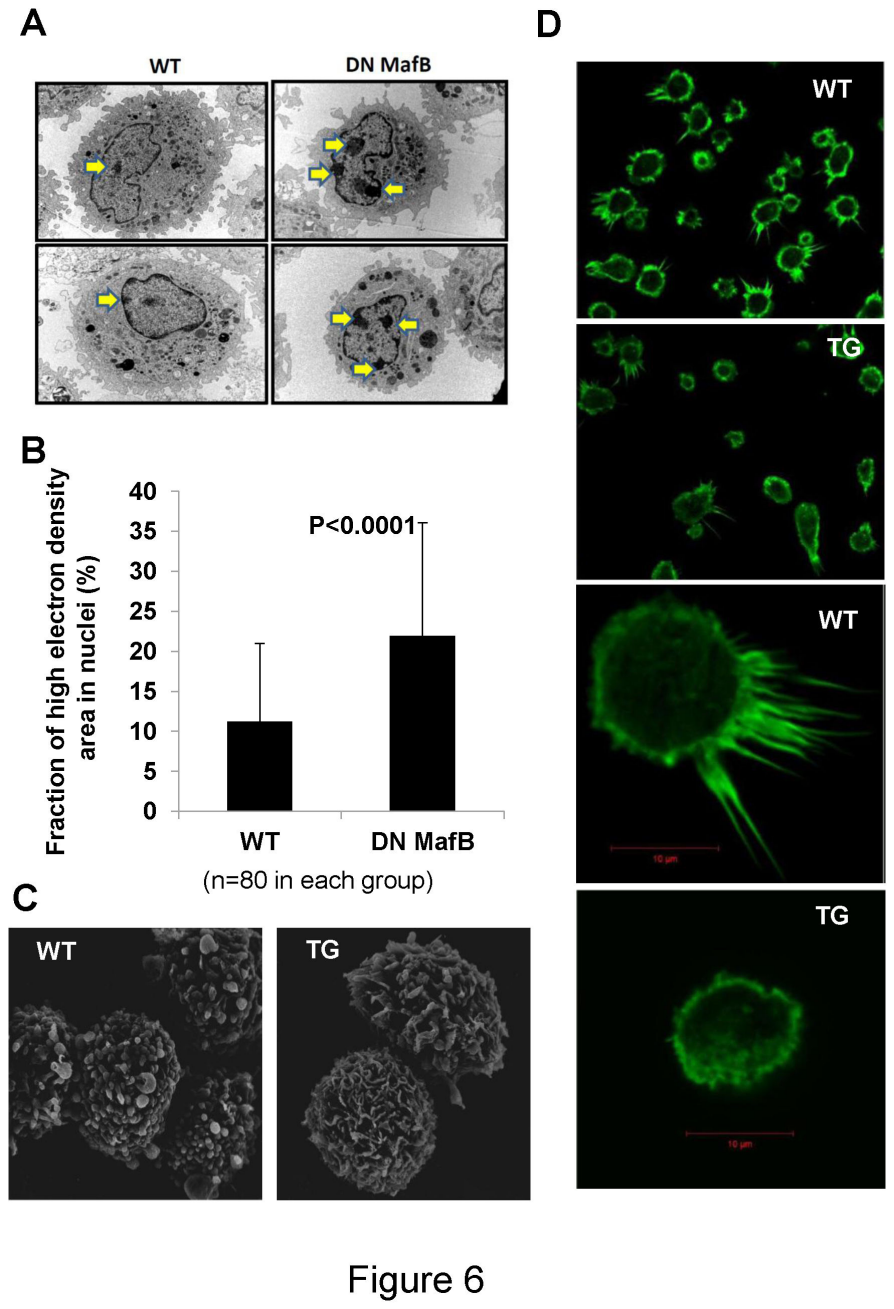
Morphological changes in alveolar macrophages (AMs) in macrophage scavenger receptor (MSR) enhancer-promoter dominant negative (DN) MafB transgenic (TG) mice. (A) Transmission electric microscopy revealed that the high electron density area (arrow) in the nucleus was increased in TG mice. Original magnification: ×7000. (B) The high electron density area in each nucleus was quantified (as described in *Materials and Methods*) and compared. The fraction of high electron density area in the nuclei of TG mice was significantly increased, compared with wild-type (WT) mice. (C) Scanning electron microscopy revealed that the morphology of pseudopods of AMs was different. In WT mice, pseudopod formations were narrow rod-like structures (‘filopodium-like’) while they were flat ruffled structures in the TG mice (‘lamellipodium-like’). Original magnification: ×8000. (D) Formation of actin filament (F-actin) was investigated using fluorescent-labeled phalloidin. In WT mice, alveolar macrophages projected more pseudopods than in TG mice. F-actin was dominantly localized in pseudopods, and fluorescent signal was stronger in the alveolar macrophages of WT mice, compared with TG mice. Top and second panel: original magnification: ×200. Third and bottom panel: original magnification: ×630. WT: wild-type mice, TG: transgenic mice.

The ultrastructure of AMs was evaluated by electron microscopy. Transmission electron microscopy (TEM) revealed that the high electron density area in the nuclei of TG mice was significantly increased, compared with WT mice (Figure 6A and B). In addition, scanning electron microscopy (SEM) revealed that the morphology of pseudopods of AMs in TG mice was different to that in WT mice. In WT mice, the pseudopods were narrow rod-like structures while in TG mice they were flat ruffled structures (Figure 6C). The formation of actin filament (F-actin) was investigated using fluorescent-labeled phalloidin. In TG mice, alveolar macrophages projected less pseudopods than in WT mice. F-actin was dominantly localized in pseudopods, and fluorescent signal was less in the AMs of TG mice compared with WT mice (Figure 6D).

### Surface markers on PMs and AMs

**Figure 7:**
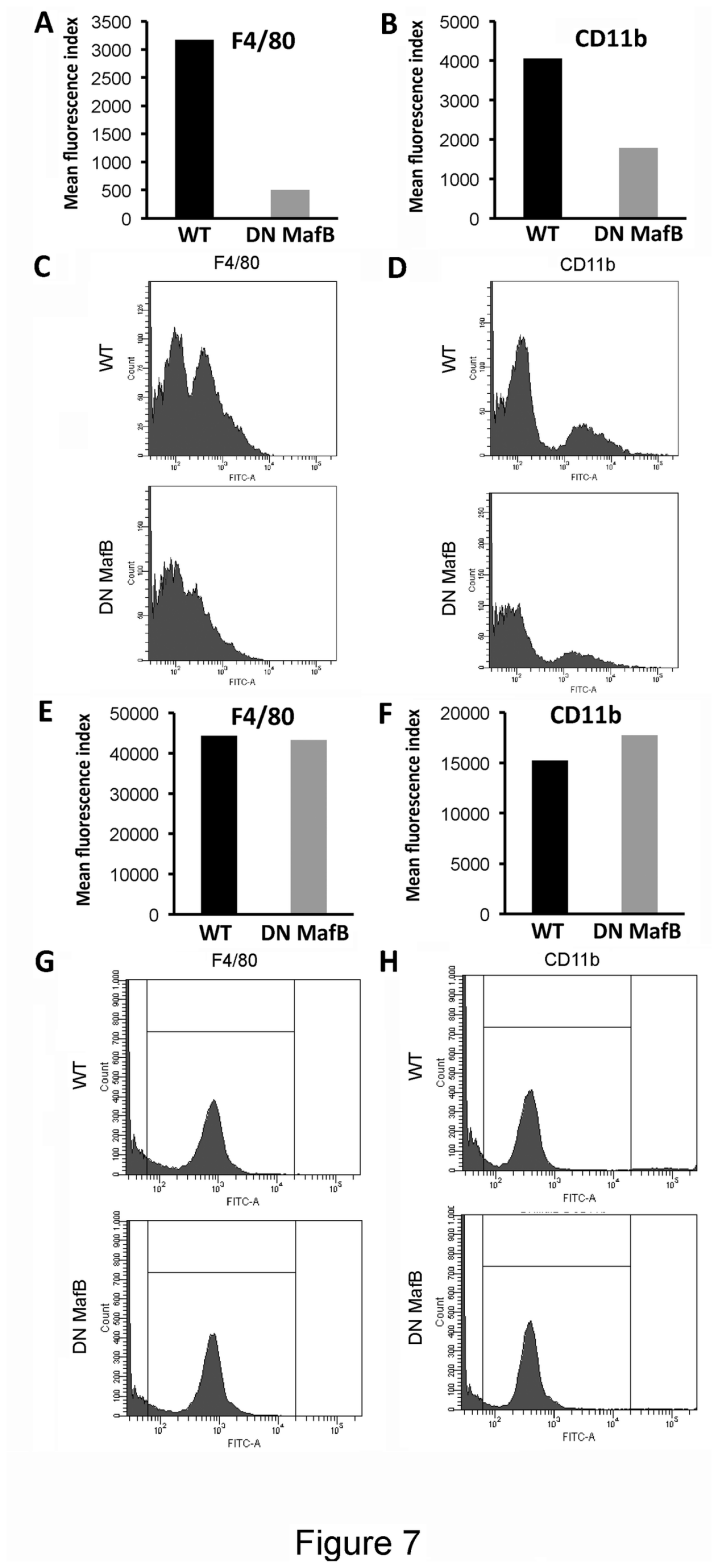
Flow cytometry of peritoneal macrophages (PMs) and alveolar macrophages (AMs). The fraction of macrophage was gated and surface markers of wild-type (WT) (black bar) and macrophage scavenger receptor (MSR) enhancer-promoter dominant negative (DN) MafB transgenic (TG) mice (gray bar) were evaluated on PMs (A & B) and AMs (E & F). Expression of F4/80, and CD11b was attenuated on PMs in TG mice, while their expression on AMs remained unchanged when compared with WT mice. The representative histogram data of three independent experiments were shown: F4/80 and CD11b expressions in PMs (C & D, respectively), or AMs (G & H, respectively).

Figure 7 shows flow cytometric observations of surface makers (F4/80 and CD11b) on PMs and AMs from WT and TG mice. Expression of both surface markers on PMs from TG mice were attenuated compared with WT mice (Figure 7A & B), while expression on AMs from TG mice remained unchanged (Figure 7E & F).

### FcR mediated-phagocytosis assay of AMs

**Figure 8:**
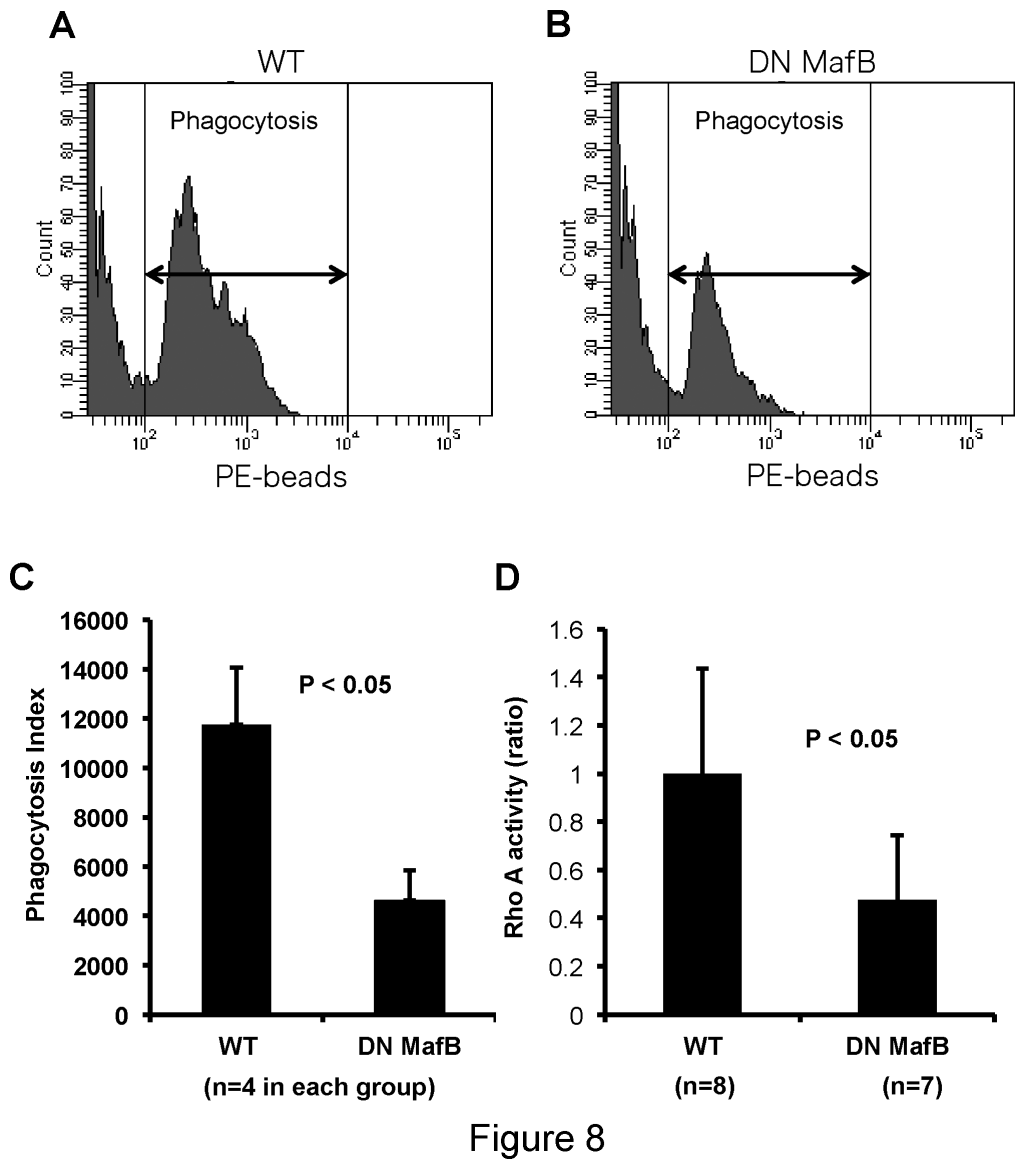
Phagocytosis assay of alveolar macrophages (AMs) in macrophage scavenger receptor (MSR) enhancer-promoter dominant negative (DN) MafB transgenic (TG) mice. (A) (B) The phagocytic capacity of TG and wild-type (WT) AMs for IgG coated phycoerythrin (PE)-labeled polystyrene beads was analyzed using flow cytometry. The representative histogram data of WT (A) and TG (B) were shown. (A) Phagocytic capacity was significantly reduced in AMs of TG mice, compared to those of WT mice. (B) Rho GTPase activity was significantly reduced in TG mice compared with WT mice.

To compare the phagocytic capacity of AMs in TG and WT mice, we performed a flow-based phagocytosis assay. The phagocytic index of IgG-coated opsonized PE-labeled beads was significantly reduced in TG mice compared with WT mice (Figure 8A–C). Rho GTPase activity, a key molecule involved in phagocytosis, was significantly reduced in the BMDMs of TG mice, compared with WT mice (Figure 8B).

## Discussion

We generated TG mice that express DN MafB capable of suppressing endogenous MafB transcription activity in macrophages (Figures 2 and 3). Numbers of AMs were significantly decreased in the TG mice compared with the WT mice (Table 1). Morphological analyses using light and electron microscopy revealed an increased fraction of high electron density area in the nuclei (heterochromatin) and an altered shape of the pseudopods on AMs in the TG mice (Figure 6). In addition, expression of F4/80, a macrophage differentiation marker, on PMs in the TG mouse was significantly reduced compared with WT mice (Figure 7). Thus, it would appear that the macrophage phenotype was altered in the TG mouse due to the lack of MafB transcription activity. Reduced phagocytic capacity of opsonized beads also suggested altered function of the macrophages in the TG mouse (Figure 8). Therefore, this study indicates an important role of MafB in the maintenance of AM number and macrophage function.

AMs are believed to play an important role in the pathogenesis of COPD, and are significantly increased in COPD patients [3]. AMs release inflammatory mediators including tumor necrosis factor-α, interleukin (IL)-1β, and IL-6 after stimulation with cigarette smoke extract (CSE). Alveolar destruction and prolonged inflammation occur via these mediators in the lungs. We previously demonstrated that MafB was upregulated in AMs in the lungs of mice with cigarette smoke-induced emphysema, suggesting a relationship between cigarette smoking and MafB expression [6]. Furthermore, we reported that the intensity of MafB immunostaining in AMs correlated with the degree of airflow limitation in human smokers, suggesting an important role for MafB in COPD [7].

To investigate the role of MafB *in vivo*, we needed to generate an advanced MafB gene-targeted animal that could live longer as MafB knockout mice die immediately after birth due to developmental anomalies in respiratory center neurons [10]. In this study we established a TG mouse that can suppress the activity of endogenous MafB by expressing DN MafB only in macrophages under the control of the MSR enhancer-promoter. In this mouse, DN MafB was detected in Kupffer cells in the liver, podocytes and Bowman capsule cells in the kidney, splenic red pulp cells in the spleen, and alveolar macrophages. This distribution is reasonable, because Kupffer cells, podocytes and splenic red pulp cells are known to have macrophage characters [20–22]. As shown in Figure 3A, repression of MafB activity in the macrophages of this TG mouse was significant, however, the survival rate of the TG mice was similar to WT mice. Therefore, this TG mouse presents a perfect model for *in vivo* experiments to investigate the role of MafB, as opposed to using MafB deficient mouse.

Comparing the phenotype of AMs between the TG and WT mice suggested an important role for MafB. First, MafB plays a role in maintaining the number of AMs *in vivo*. In COPD patients, programmed cell death is observed in various cells and influences the viability of cells in the lung [23–25]. However, the AMs of smokers reportedly survive longer than those of non-smokers [26,27]. We previously demonstrated that over-expression of MafB enhanced cell viability following treatment with CSE as well as significantly inhibiting caspase-3 activity and reducing macrophage apoptosis [6]. In this study, the numbers of AMs were significantly decreased in the TG mice. This reduced number of AMs may be attributable to enhanced apoptosis due to the inhibition of endogenous MafB activity. The findings of elevated caspase-3 expression in AMs and an increased number of apoptosing cells in the spleen strongly support this hypothesis.

Second, MafB may play a role in regulating transcription activity via chromosomal structural changes *in vivo*. In the TG mice, the fraction of high electron density area in the nuclei (heterochromatin) was increased, compared with WT mice. Structural changes in chromosomes is an important mechanism of gene regulation [28], and the association of specific genes with heterochromatin alters the efficacy of gene expression [29]. Thus, increasing heterochromatin by suppressing endogenous MafB activity may result in the reduced gene expression in AMs.

Third, MafB may play a role in regulating the differentiation of macrophages *in vivo*. MafB is expressed only in macrophage lineage cells derived hematopoietic cells. Also, MafB is known to be expressed in some non-hematopoietic cells, such as podocytes [13]. F4/80 is a specific cell surface marker for murine macrophages [30,31], and is known to be a member of the epidermal growth factor-seven-transmembrane-domain family. As the precursor of tissue macrophages, blood monocytes express less F4/80 than mature macrophages [32]. CD11b is expressed on the surface of monocytes, macrophages, NK cells, and granulocytes. AMs express less CD11b than their monocytic precursors [33]. Moriguchi et al. reported that F4/80 expression in macrophages was attenuated in a homozygous mutant of MafB knockout mice [13], while Aziz et al. reported that F4/80 expression in macrophages remained unchanged in MafB deficient mice [34]. In the present study, F4/80 and CD11b expression in PMs of TG mice was suppressed compared with WT mice, while their expression was not changed in AMs in TG mice. In the lungs of granulocyte macrophage colony stimulating factor (GM-CSF) deficient mice, many AM functions are impaired and alveolar proteinosis is developed due to the reduction of PU.1, a master regulator of macrophage differentiation [35]. However, cell differentials in the peripheral blood are normal [36], and macrophage dysfunction in other organs has not been reported in GM-CSF deficient mice. Thus, the AM differentiation process is suggested to be specific and different from that of macrophages from other organs. The difference in surface marker expression between PMs and AMs may be attributable to this specific differentiation process in AMs. As macrophage differentiation is regulated by other transcription factors, such as PU.1 and interferon regulatory factor-8 [35,37], and not only MafB, we need to assess how the activity of these transcription factors affects macrophage function in the TG mice.

Fourth, MafB may play a role in regulating macrophage phagocytosis by changing the cytoskeletal organization. In WT mice, the structure of pseudopods on the majority of AMs had a ‘filopodia-like’ shape while in TG mice they had a ‘lamellipodium-like’ shape. The structure of pseudopods depends heavily on cytoskeletal factors, such as actin and microtubules [38]. MafB deficient mice reportedly have abnormal actin formation [34]. During phagocytosis an orderly change of cytoskeletal conformation, including actin formation, is required [39]. As shown in Figure 6D, actin filament formation was attenuated in the pseudopods of AMs in TG mice. In addition, the Rho GTPase activity, a molecule regulating cytoskeletal organization during phagocytosis, was significantly reduced in TG mice, compared with WT mice [40]. Thus, this structural change in the pseudopods may affect the phagocytic capacity of AMs in TG mice.

In conclusion, the present study demonstrated that MafB regulates the macrophage phenotype with respect to the number of alveolar macrophages, the nuclear compartment, cellular shape, surface marker expression, and phagocytic function. Furthermore, the MSR-DN MafB TG mouse model has the potential to contribute to our understanding of the pathogenesis of respiratory diseases including COPD.
